# Effects of morning versus evening exercise on vascular function in older adults with type 2 diabetes: a randomised controlled trial

**DOI:** 10.3389/fendo.2026.1976870

**Published:** 2026-10-16

**Authors:** Zhiying Ma, Yanyu Lin, Zheng Zhu, Zhen-Bo Cao

**Affiliations:** 1 School of Exercise and Health, Key Laboratory of Exercise and Health Sciences of Ministry of Education, Shanghai University of Sport, Shanghai, China; 2 AUT Sport Research Institute New Zealand (SPRINZ), Auckland University of Technology, Auckland, New Zealand

**Keywords:** arterial stiffness, blood pressure, endothelial function, exercise timing, T2DM, vascular function

## Abstract

**Background:**

Cardiovascular disease is a common complication of type 2 diabetes mellitus (T2DM), with increased risk closely associated with vascular dysfunction. Exercise is an effective strategy for improving vascular health, but whether exercise timing influences long-term vascular adaptations in patients with T2DM remains unclear. In this study, we investigated the effects of morning versus evening exercise on vascular function in older adults with T2DM.

**Methods:**

Sixty-six older adults with T2DM were randomised to morning exercise, evening exercise, or control (n = 22 each). The exercise groups received 12 weeks of online moderate-intensity aerobic and resistance exercises, three times per week. The morning group exercised from 9:00 to 10:10, and the evening group from 19:00 to 20:10. The control group maintained their usual lifestyle and attended weekly health education sessions without structured exercise. Blood pressure, brachial-ankle pulse wave velocity (baPWV), flow-mediated vasodilation (FMD) and plasma nitric oxide (NO) levels were assessed at baseline and post-intervention. Outcomes were analysed using two-way repeated-measures analysis of variance, with exploratory analyses directly comparing the two exercise groups.

**Results:**

Fifty-eight participants were included in the final analysis (morning, n = 20; evening, n = 19; control, n = 19). Significant group × time interactions were observed for FMD, baPWV, systolic blood pressure (SBP), mean arterial pressure (MAP), and plasma NO (all p < 0.05). Both exercise groups increased FMD and reduced SBP, MAP, and baPWV from baseline (all p < 0.05). Plasma NO increased significantly following the evening exercise (p = 0.003), whereas no significant changes were observed following the morning exercise (p = 0.722). At post-intervention, the evening group exhibited lower MAP (p = 0.012) and higher FMD (p = 0.019) and NO levels (p = 0.012) than controls. Exploratory analyses showed a greater baPWV reduction with evening exercise (group × time interaction, p = 0.034). The increase in plasma NO was numerically greater with evening exercise, although the interaction was not statistically significant (p = 0.051).

**Conclusions:**

Exercise timing may influence vascular adaptations in older adults with T2DM, with evening exercise potentially offering additional benefits, particularly for arterial stiffness.

**Clinical trial registration:**

https://www.chictr.org.cn/showproj.html?proj=200163, identifier ChiCTR2300073737.

## 1 Introduction

Type 2 diabetes mellitus (T2DM) is a chronic metabolic disorder characterised by hyperglycaemia caused by insulin deficiency and peripheral insulin resistance (1). Cardiovascular disease (CVD) is the most common and serious complication of T2DM, with affected individuals facing a 2–4-fold higher risk of CVD than those without diabetes (2). CVD contributes to nearly half of all deaths in this population (3), highlighting the need for integrated cardiovascular risk management and glycaemic control.

The increased cardiovascular risk observed in patients with T2DM is closely associated with vascular dysfunction. T2DM is a major risk factor for vascular disease, and multiple vascular comorbidities substantially impair prognosis and complicate treatment (4). Vascular function, including vasodilation, arterial compliance, and blood flow regulation, is frequently impaired in individuals with T2DM (5–7). When imbalanced homeostasis is characterised by abnormal glucose and lipid metabolism, the activation of the renin–angiotensin–aldosterone system and sympathetic nervous system directly or indirectly causes widespread vascular damage throughout the body, leading to the development of pan-vascular complications in T2DM (4).

Within this broader vascular pathology, endothelial dysfunction is recognised as an early stage in the onset and progression of cardiovascular complications in patients with T2DM (8). Endothelial cells synthesise several vasoactive substances, with nitric oxide (NO) serving as a key vasodilator crucial for maintaining vascular homeostasis (9). Endothelial dysfunction is characterised by reduced NO production and bioavailability, leading to impaired vasodilation, increased arterial stiffness, and an elevated cardiovascular risk.

Exercise is an effective intervention for enhancing endothelial function in individuals with T2DM (10) since it promotes beneficial physiological adaptations in the cardiovascular system. Exercise enhances blood flow, thereby increasing shear stress on endothelial cells (11), which promotes NO synthesis and bioavailability (12) while simultaneously reducing oxidative stress and the expression of proinflammatory molecules (13), collectively contributing to improved endothelial function. Current exercise guidelines recommend the type, intensity, frequency, duration, and progression of exercise for individuals with T2DM. However, clear guidance is lacking on whether exercise timing within the day should be considered in exercise planning (14).

Exercise timing may matter because cardiovascular function varies over 24 h under the influence of the endogenous circadian system (15). In the early morning, sympathetic nervous system activity increases, accompanied by elevated plasma levels of cortisol, catecholamines, angiotensin II, platelet aggregates, and plasminogen activator inhibitor-1 (PAI-1) (16). Concurrently, heart rate and blood pressure rise, serum NO levels decrease (17), and flow-mediated vasodilation (FMD) of the brachial artery is attenuated compared with other times of the day (18). These early morning alterations are thought to contribute to the well-documented peak of cardiovascular events during this period (19). Conversely, during the early evening, most of these physiological patterns reverse, and cardiovascular risk appears to be lower owing to the complex interplay between endogenous circadian rhythms, environmental factors, and behavioural patterns (16). Exercise can act as a nonphotic zeitgeber (20) and may help resynchronise circadian rhythms and reduce cardiovascular risk (21).

Previous studies have reported inconsistent findings regarding the effects of exercise timing on endothelial function. Jones et al. (22) reported that moderate-intensity interval exercise performed in the morning had no significant effect on FMD, whereas the same exercise performed in the afternoon significantly reduced FMD. Ballard et al. (23) observed no significant changes in FMD after morning or afternoon exercise. However, afternoon exercise led to a marked increase in shear rate, which is a key stimulus for NO production. While most studies examined acute endothelial responses, Shen et al. (24) demonstrated in a 12-week aerobic training intervention in sedentary adult men that compared with morning exercise, evening exercise produced superior long-term improvements in carotid artery haemodynamics and vascular compliance. To date, most studies examining the effects of exercise timing on endothelial function have been limited to acute interventions in healthy young men. Whether long-term exercise interventions at different times of the day elicit differential vascular adaptations in populations with elevated cardiovascular risk, such as patients with T2DM, remains largely unexplored.

In this study, we compared the effects of a 12-week combined aerobic and resistance exercise intervention performed in the morning versus the evening on endothelial function, arterial stiffness, and blood pressure in individuals with T2DM. We hypothesised that evening exercise would lead to greater improvements in cardiovascular outcomes than morning exercise.

## 2 Materials and methods

### 2.1 Participants

This was a 12-week randomised controlled study; 66 participants were recruited from the diabetes clinic of a hospital in Shanghai through posted advertisements and via diabetes awareness campaigns held in the community of Yangpu District. All laboratory assessments were performed at the Shanghai University of Sport. Eligibility was assessed during an initial screening interview, which included self-reported medical history and completion of the Physical Activity Readiness Questionnaire (PAR-Q). Participants were eligible if they had T2DM diagnosed according to the American Diabetes Association criteria, were aged 60–75 years, and reported not meeting the recommended levels of ≥ 150 min/week of moderate-intensity or ≥75 min/week of vigorous-intensity exercise during the preceding 3 months (25). Participants were excluded if the screening identified serious cardiovascular disease, a history of stroke, cancer, psychiatric disorders, musculoskeletal impairments that restricted exercise participation, or severe diabetes-related complications that could compromise exercise safety. For participants taking prescription medications, medication use was recorded at baseline, and they were instructed to maintain their usual regimens throughout the intervention, with any changes assessed at the follow-up.

After baseline assessments, eligible participants were randomly assigned in a 1:1:1 ratio to the morning exercise group, evening exercise group, or control group using block randomisation with a block size of six. An independent statistician generated the randomisation sequence, which was implemented through an independent online computerised randomisation system (https://www.sealedenvelope.com/simple-randomiser/v1/lists). Because the training time was the core component of the intervention, the participants and fitness coaches could not be blinded to group allocation. However, outcome assessors and data analysts were blinded to group allocation, and fitness coaches were kept separate from assessment. Group allocation was revealed only after all statistical analyses had been completed. Participants were excluded if they dropped out or completed less than 80% of the training schedule. This study was approved by the Ethics Committee of the Shanghai University of Sport (102772022RT078) and registered in the Chinese Clinical Trial Registry (ChiCTR2300073737). Written informed consent was obtained from all participants.

### 2.2 Exercise intervention

All participants were instructed not to alter their dietary habits throughout the intervention period. Participants in the control group were instructed to maintain their usual lifestyle and attend a weekly 30-min online health education session. The exercise groups trained in the morning (09:00–10:10) or evening (19:00–20:10), according to their assigned training time slots. Each group completed a 12-week moderate-intensity combined aerobic and resistance training programme delivered via Tencent Meeting. The aerobic component consisted of a music-based aerobic exercise adapted from the video materials accompanying the Comprehensive Geriatric Intervention Program (26). The routine involved rhythmic marching and stepping with coordinated upper- and lower-body movements. The resistance training component was performed in a circuit format and consisted of 10 elastic-band exercises: reverse fly, chest press, front raise, biceps curl, lateral trunk flexion, single-leg press, hip abduction, standing heel raise, squat, and lateral walk exercises. Participants completed two circuits per session, with one set of each exercise performed sequentially in each circuit, 30 s of rest between exercises, and 2 min of rest between circuits.

Before the intervention, participants in the exercise groups received in-person instruction on proper exercise technique from a certified exercise coach. During the intervention period, the participants accessed prerecorded exercise videos through a designated virtual meeting room and followed routines under remote supervision. The supervising coach monitored participants throughout each session via Tencent Meeting. Participants were required to keep their cameras on for the entire session so the coach could verify their participation and completion of the prescribed exercises. The 12-week intervention included a 1-week adaptation phase that focussed on movement instruction, followed by 11 weeks of formal training. Exercise sessions were held three times per week, with at least 48 h between sessions. Each 70-min session included a 5-min warm-up, approximately 30 min of aerobic exercise, 30 min of resistance training, and a 5-min cool-down. The warm-up consisted of dynamic mobility exercises, whereas the cool-down consisted of static stretching.

During the intervention period, exercise intensity increased progressively. For aerobic exercise, the intensity was adjusted by increasing the playback speed of the exercise videos by 20% every 4 weeks. Heart rate was monitored using a wrist-worn device (Vantage V, Polar Electro Oy, Kempele, Finland) to guide exercise intensity within the prescribed range, and ratings of perceived exertion (RPE) were obtained after each session using the Borg 6–20 scale. For resistance exercise, elastic bands with appropriate resistance levels were selected based on each participant’s upper- and lower-limb strength, and the resistance was increased by 5 pounds every 4 weeks. **Table 1** presents the exercise intensity progression scheme.

**Table 1:** Exercise loading protocol.

Exercise modality	Time	Exercise load
Aerobic exercise	1–4 week	40%-50% HRR
	5–8 week	50%-60% HRR
	9–12 week	50%-60%HRR
Resistance exercise	1–4 week	2 sets*15 reps*10 movements
	5–8 week	2 sets*12 reps*10 movements
	9–12 week	2 sets*12 reps*10 movements

HRR, Heart Rate Reserve.

### 2.3 Outcome assessments

All outcome measures were assessed at baseline and after the 12-week intervention. In both exercise groups, post-intervention assessments were conducted 48–72 h after the final exercise session to minimise the potential influence of acute exercise and short-term carryover effects. During the 24 h before each assessment, participants were instructed to abstain from alcohol and caffeine, refrain from vigorous physical activity, and maintain their usual sleep schedule. Participants also fasted for at least 12 h and postponed their scheduled morning medications until all assessments had been completed.

### 2.4 Anthropometry

Height and weight were measured using a stadiometer (TANITA DC-250, Japan). Body fat percentage was measured using a dual-energy X-ray absorptiometry (DXA) device (DXA Prodigy, GE Lunar Corp., Madison, WI, USA). Body mass index was calculated as body mass (kg)/height squared (m²).

### 2.5 Brachial blood pressure

Brachial systolic blood pressure (SBP) and diastolic blood pressure (DBP) were measured with participants seated using an automated oscillometric device (HEM-907-E; Omron, Tokyo, Japan). Before the first measurement, participants rested quietly in a seated position for 5 min. Two measurements were taken at 1-min intervals. If either SBP or DBP differed by > 5 mmHg between the first two measurements, a third measurement was performed. The mean of the two closest readings was used as the final SBP and DBP values for analysis. Mean arterial pressure (MAP) was calculated using the formula: MAP = DBP + [1/3 (SBP − DBP)].

### 2.6 Flow-mediated vasodilation

All FMD measurements were performed at the same time of the day. Participants lay in a supine position for 20 min in a quiet, temperature-controlled laboratory environment (approximately 25 °C). FMD measurements were performed on the right upper arm, with the participant’s right arm extended and positioned at an angle of approximately 80°from the torso. A pneumatic cuff was positioned 2 cm distal to the olecranon of the forearm.

A 10 MHz linear array probe connected to a high-resolution Doppler ultrasound machine (LOGIQ e, GE Healthcare, Pittsburgh, PA, USA) was used to image the brachial artery. Once an optimal image was obtained, the probe was held stable, and the ultrasound parameters were adjusted to optimise the longitudinal B-mode image of the lumen-arterial wall interface.

In accordance with published FMD guidelines (27), B-mode and Doppler images were obtained to determine the brachial artery diameter and blood velocity, respectively. Baseline brachial artery diameter images were captured for 120 s before cuff inflation, together with measurements of baseline blood flow velocity. The cuff was then inflated to 240 mmHg and maintained for 5 min to occlude blood flow. During cuff inflation, the ultrasound probe was kept steady to ensure consistent measurement positions before and after inflation. Longitudinal images of the brachial artery were recorded continuously for 30 s before and 180 s after cuff deflation (28).

All images were analysed offline using automated edge detection software (Brachial Analyser, Medical Imaging Applications, Coralville, IA, USA), which is largely independent of investigator bias. The software automatically synchronised the image selection with the peak of the R-wave on the electrocardiogram. Frames that did not meet the required quality standards (confidence threshold < 70%) were excluded. The formula for calculating FMD was FMD% = (Peak Diameter−Baseline Diameter)/Baseline Diameter ×100.

### 2.7 Brachial-ankle pulse wave velocity

BaPWV and ankle–brachial index (ABI) were measured simultaneously with participants in the supine position after at least 20 min of rest using an automatic arterial stiffness analyser (BP-203RPEIII, Omron, Osaka, Japan). Cuffs were placed around the arms and ankles, electrocardiographic electrodes were positioned on both wrists, and a heart-sound microphone was placed at the left sternal edge. The analyser simultaneously recorded pulse volume waveforms, phonocardiogram, and arterial blood pressure in both arms and ankles, and automatically calculated baPWV and ABI values. The left and right baPWV values were averaged for analysis in this study.

### 2.8 Biochemical analyses

Blood samples were collected after 12 h of fasting and immediately centrifuged at 3,000 rpm (maintained at 4 °C) for 15 min to separate the components. Serum and plasma samples were aliquoted and stored in an ultra-low-temperature freezer at −80 °C until subsequent analysis. Glycated haemoglobin and blood glucose levels were measured by an independent commercial pathology laboratory (Jince Medical Testing Laboratory, Shanghai, China). NO levels were indirectly assessed by measuring NO metabolites, nitrite (NO_2_−) and nitrate (NO3−), using an assay kit (A012; Nanjing Jiancheng Bioengineering Institute, Nanjing). The concentrations were determined using spectrophotometric analysis.

### 2.9 Chronotype and physical activity assessment

Chronotype was assessed at baseline using the Chinese version of the Morningness-Eveningness Questionnaire (MEQ) (29). Participants were classified as morning (59–86), intermediate (42–58), or evening types (16–41) according to their total scores (30).

Baseline physical activity was assessed using hip-worn triaxial accelerometers (wGT3X-BT; ActiGraph LLC, Pensacola, FL, USA) for seven consecutive days. Data were analysed in 60-s epochs using activAnalyzer (31). Accelerometer analyses required ≥4 valid days (≥8 h of wear time/day), including ≥3 weekdays and ≥1 weekend day. Moderate-to-vigorous physical activity [MVPA; ≥1952 vertical-axis counts/min (32)] was averaged across all valid days (min/day).

### 2.10 Statistical analysis

The sample size calculation was based on FMD, the primary vascular function outcome. Based on previous evidence (33), 30 participants were required to detect a group × time interaction in a repeated-measures ANOVA, assuming an effect size of 0.40 (α = 0.05; 1−β = 0.95) (G*Power version 3.1.9.7, Heinrich Heine University, Düsseldorf, Germany). Participants were included in the final analysis if they completed ≥ 80% of intervention sessions, had complete baseline and post-intervention outcome data, and reported no medication changes. Missing data were not imputed.

Analyses were conducted using SPSS version 25.0 (IBM Corp., Armonk, NY, USA). Continuous variables are expressed as mean ± standard deviation. Normality of distribution was assessed using the Kolmogorov–Smirnov test. One-way analysis of variance (ANOVA) was used to compare baseline continuous variables among the groups, and the chi-square or Fisher’s exact tests were applied for baseline categorical variables. Changes in vascular function, blood biomarkers, and anthropometric indices were analysed using 3 (group: morning exercise, evening exercise, and control) × 2 (time: baseline and post-intervention) two-way repeated-measures ANOVAs, with group as the between-subjects factor and time as the within-subjects factor. To directly compare changes between morning and evening exercise, supplementary exploratory 2 (group: morning exercise and evening exercise) × 2 (time: baseline and post-intervention) two-way repeated-measures ANOVAs were subsequently performed. The group × time interaction was examined to determine whether changes from baseline to post-intervention differed between the two exercise groups. *Post hoc* comparisons were performed using the least significant difference test. Statistical significance was set at p < 0.05.

## 3 Results

### 3.1 Participant characteristics

**Figure 1** shows participant recruitment and allocation. Of the 593 individuals who initially contacted us, 388 did not meet the inclusion criteria, 96 were unwilling to participate, and 43 were excluded for other reasons. We enrolled 66 eligible participants and randomly assigned them to three groups (n = 22 each). No exercise-related adverse events occurred during the intervention. Six participants discontinued the intervention: three for health-related reasons and three for family-related reasons. Additionally, two participants in the control group were excluded: one because of substantial lifestyle change and the other because of medication changes during the intervention. No participants were excluded because of exercise adherence below 80%. Ultimately, 58 participants were included in the final analysis. Among participants in the exercise groups, the attendance rate was 95.3 ± 6.9% in the morning exercise group and 97.5 ± 4.7% in the evening exercise group. RPE scores ranged from 11 to 15 in both exercise groups. **Table 2** shows the participants’ baseline characteristics. Among these, only lipid-lowering medication use differed significantly among the three groups (p = 0.028).

**Figure 1:**
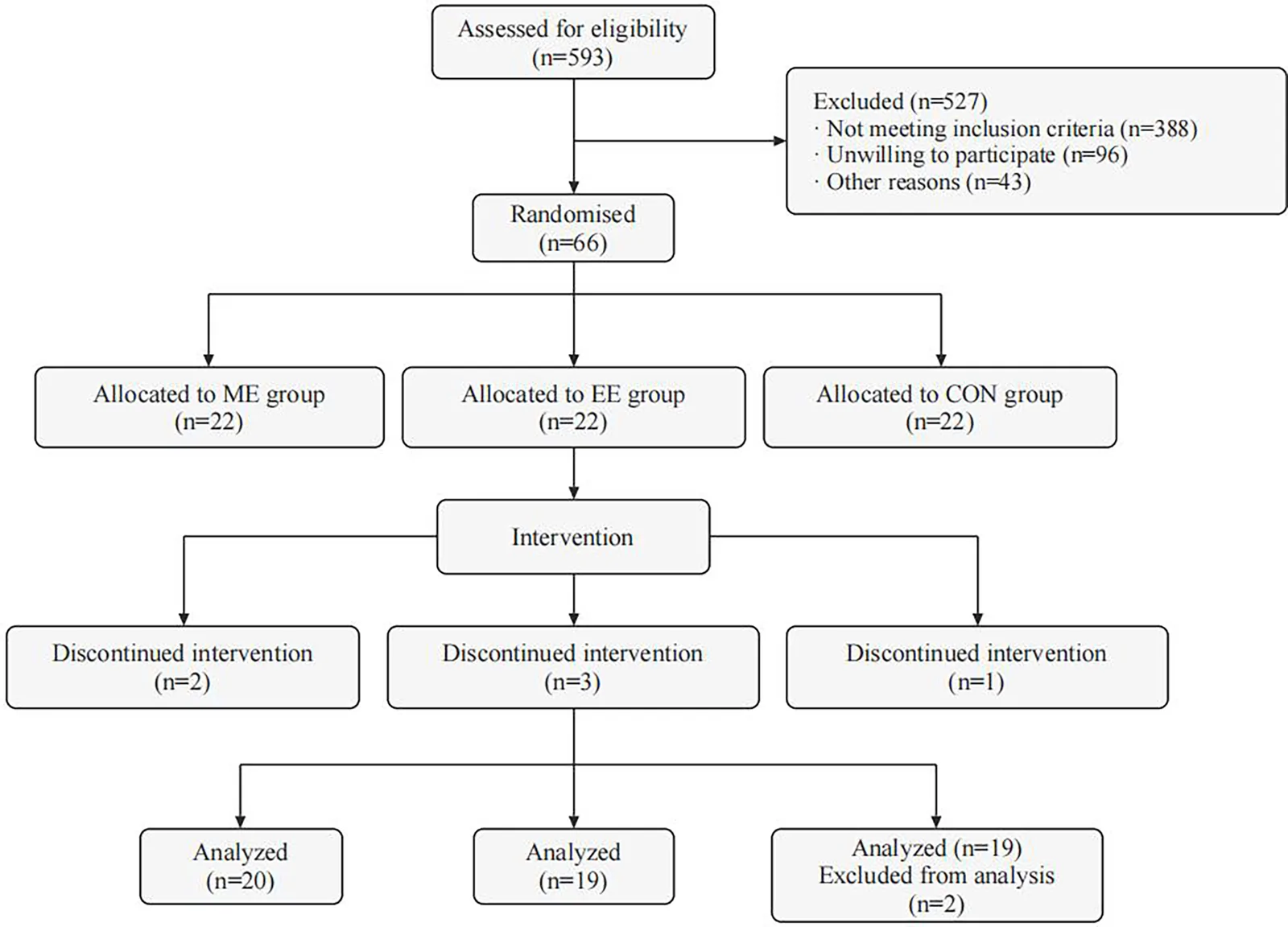
Flowchart of participant inclusion.

**Table 2:** Characteristics of groups.

Characteristic	All (n=58)	CON (n=19)	ME (n=20)	EE (n=19)	p
Age (years)	67.21 ± 3.87	66.95 ± 4.24	67.15 ± 3.68	67.53 ± 3.86	0.899
Sex(M/F)	29/29	11/8	10/10	8/11	0.623
Height(m)	1.63 ± 0.08	1.63 ± 0.09	1.64 ± 0.07	1.63 ± 0.08	0.931
Weight(kg)	66.78 ± 11.48	66.53 ± 13.17	68.02 ± 10.75	65.75 ± 10.91	0.826
BMI(kg/m^2^)	24.95 ± 3.16	24.94 ± 3.24	25.24 ± 3.08	24.67 ± 3.31	0.859
Body fat percentage(%)	31.42 ± 5.83	30.82 ± 4.62	32.16 ± 6.45	31.25 ± 6.44	0.771
Waist circumference (cm)	91.17 ± 8.16	90.47 ± 8.48	92.58 ± 7.99	90.40 ± 8.26	0.645
Duration of diabetes (years)	11.09 ± 8.45	11.89 ± 10.68	9.55 ± 7.57	11.89 ± 6.90	0.612
HbA1c(%)	7.28 ± 1.33	7.35 ± 1.16	7.07 ± 1.28	7.43 ± 1.57	0.687
FBG (mmol/L)	7.91 ± 2.48	7.69 ± 2.03	7.29 ± 2.23	8.68 ± 3.01	0.213
Hypertension, n(%)	25 (43%)	6 (32%)	9 (45%)	10 (53%)	0.454
Macrovascular complications, n(%)	9 (16%)	3 (16%)	3 (15%)	3 (16%)	1.000
Microvascular complications, n(%)	7 (12%)	2 (11%)	3 (15%)	2 (11%)	1.000
Smoking, n(%)	17 (29%)	5 (26%)	7 (35%)	5 (26%)	0.818
Oral antidiabetic medication, n(%)	46 (79%)	15 (79%)	15 (75%)	16 (84%)	0.918
Insulin, n(%)	8 (14%)	2 (11%)	3 (15%)	3 (16%)	1.000
Lipid-lowering medication, n(%)	10 (17%)	4 (21%)	6 (30%)	0 (0%)	0.028
Antihypertensive medication, n(%)	25 (43%)	6 (32%)	8 (40%)	11 (58%)	0.270
Hormone replacement medication, n(%)	4 (7%)	1 (5%)	1 (5%)	2(11%)	0.838
Anti-platelet medication, n(%)	7 (12%)	2 (11%)	2 (10%)	3 (16%)	0.889
No medication	2 (3%)	1 (5%)	0 (0%)	1 (5%)	0.540
MVPA (min/day)	10.98 ± 5.15	11.53 ± 4.23	11.15 ± 4.98	10.29 ± 6.44	0.870
MEQ score	65.1 ± 7.59	66.6 ± 8.3	66.5 ± 7.1	62.0 ± 6.9	0.097
Chronotype, n (%)					0.079
Morning type	49 (84%)	17 (89%)	19 (95%)	13 (68%)	
Intermediate type	9 (16%)	2 (11%)	1 (5%)	6 (32%)	

Data are presented as mean ± SD or n (%). BMI, Body mass index; CON, Control Group; ME, Morning Exercise Group; EE, Evening Exercise Group; HbA1c, glycated haemoglobin; FBG: Fasting Blood Glucose; MVPA, moderate-to-vigorous physical activity; MEQ, Morningness–Eveningness Questionnaire.

### 3.2 Effects of morning and evening exercise on FMD

**Table 3** presents baseline and post-deflation diameters, absolute dilation, and FMD% before and after the intervention; **Figure 2a** illustrates changes in FMD. Both the main effect of time [F(1, 54) = 17.169, p < 0.001, partial η^2^ = 0.241] and the group × time interaction [F(2, 54) = 4.066, p = 0.023, partial η^2^ = 0.131] were significant. *Post hoc* analysis showed that FMD increased from baseline in both the morning (p = 0.001) and evening (p < 0.001) exercise groups. Post-intervention FMD was higher in the evening than in the control group (p = 0.019). Exploratory analysis limited to the two exercise groups revealed no significant group × time interaction for FMD [F(1, 36) = 0.097, p = 0.757].

**Table 3:** Brachial artery diameter and FMD at baseline and post-intervention.

Outcome	CON		ME		EE	
	Pre	Post	Pre	Post	Pre	Post
Baseline brachial artery diameter (mm)	4.34 ± 0.70	4.20 ± 0.58	4.37 ± 0.88	4.30 ± 0.71	4.10 ± 0.72	4.28 ± 0.74
Post-deflation diameter (mm)	4.57 ± 0.70	4.43 ± 0.62	4.59 ± 0.91	4.56 ± 0.75	4.32 ± 0.73	4.58 ± 0.77
Absolute dilation (mm)	0.24 ± 0.06	0.23 ± 0.06	0.22 ± 0.11	0.27 ± 0.10	0.22 ± 0.06	0.30 ± 0.09
FMD (%)	5.57 ± 1.66	5.54 ± 1.19	5.04 ± 2.59	6.30 ± 2.23	5.49 ± 1.80	7.16 ± 2.02

Data are presented as mean ± SD. ME, morning exercise group; EE, evening exercise group; CON, control group; FMD, flow-mediated vasodilation.

**Figure 2:**
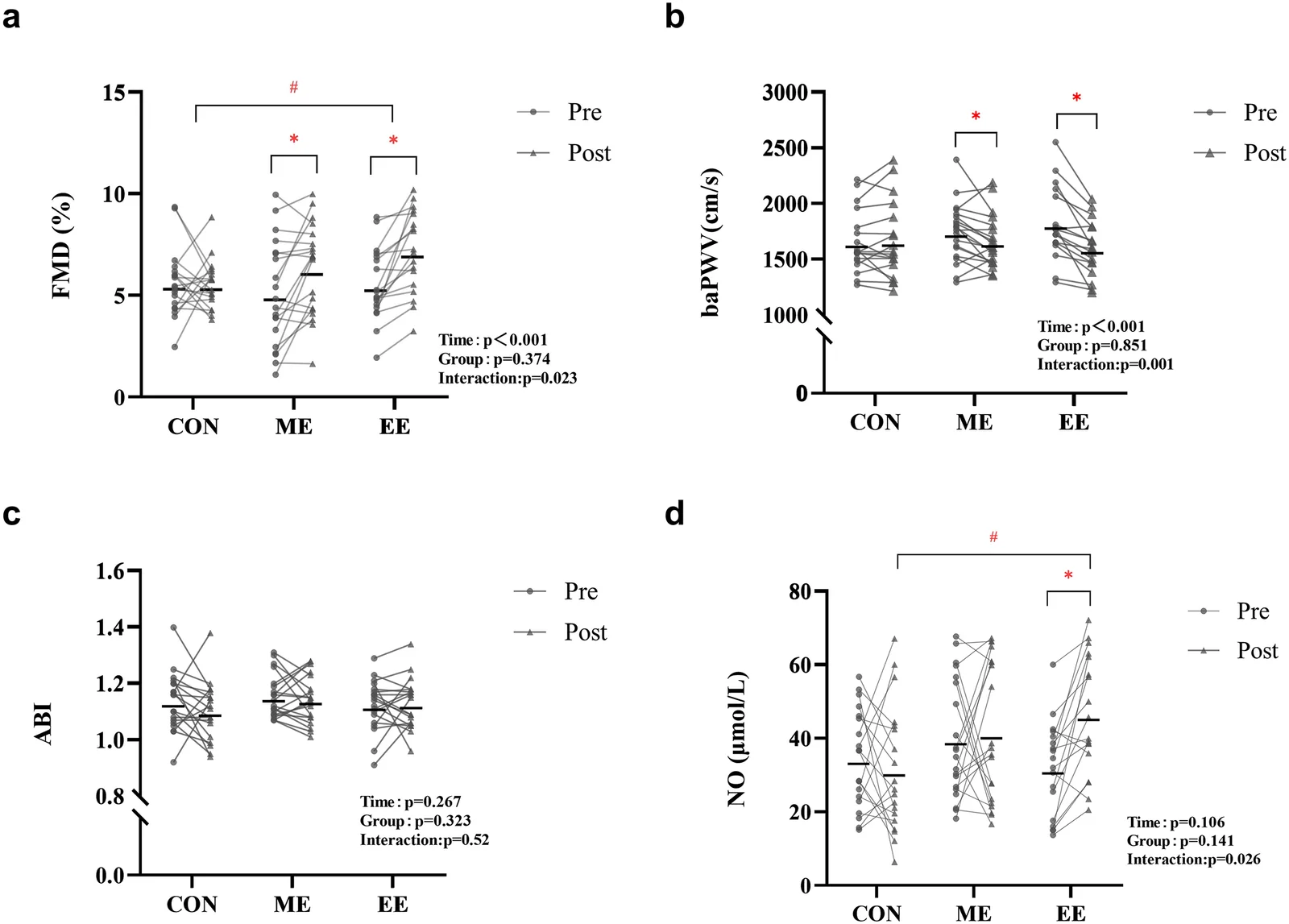
Changes in vascular outcomes following the 12-week intervention. **(a)** Flow-mediated vasodilation (FMD); **(b)** brachial–ankle pulse wave velocity (baPWV); **(c)** ankle–brachial index (ABI); and **(d)** plasma nitric oxide (NO) levels. *indicates a significant within-group difference from baseline (p < 0.05); ^#^indicates a significant difference from the control group at post-intervention (p < 0.05). CON, control group; ME, morning exercise group; EE, evening exercise group.

### 3.3 Effects of morning and evening exercise on arterial stiffness

Changes in baPWV and the ankle-brachial index are shown in **Figures 2b, c**. A significant main effect of time [F(1, 52) = 18.899, p < 0.001, partial η^2^ = 0.267] and a significant group × time interaction [F(2, 52) = 8.219, p = 0.001, partial η^2^ = 0.240] were observed for baPWV. *Post hoc* analysis indicated that baPWV significantly decreased from baseline in both the morning (from 1747.0 ± 262.1 to 1657.8 ± 234.7 cm/s, p = 0.023) and evening (from 1818.2 ± 333.4 to 1597.1 ± 250.6 cm/s, p < 0.001) groups, whereas no significant change was observed in the control group (from 1654.8 ± 278.1 to 1665.9 ± 347.4 cm/s, p = 0.782). The exploratory two-group analysis revealed a significant group × time interaction for baPWV [F(1, 35) = 4.869, p = 0.034], with a greater reduction in the evening than in the morning exercise group (−221.1 vs. −89.2 cm/s). However, no significant differences in the ankle–brachial index were observed between or within the groups across the intervention period.

### 3.4 Effects of morning and evening exercise on plasma NO

**Figure 2d** shows changes in NO levels. A significant group × time interaction was observed [F(2, 54) = 3.906, p = 0.026, partial η^2^ = 0.126]. *Post hoc* analysis showed an increase from baseline in the evening exercise group (from 31.75 ± 12.90 to 46.30 ± 16.16 μmol/L, p = 0.003), whereas no significant changes in the morning (from 39.73 ± 16.18 to 41.32 ± 19.01 μmol/L, p = 0.722) or control (from 34.39 ± 13.48 to 31.24 ± 17.24 μmol/L, p = 0.493) groups were observed. Post-intervention NO levels were higher in the evening than in the control group (p = 0.012). In the exploratory two-group analysis, the increase in NO was numerically greater in the evening than in the morning exercise group (+14.55 vs. +1.59 μmol/L), although the interaction did not reach statistical significance [F(1, 36) = 4.064, p = 0.051].

### 3.5 Effects of morning and evening exercise on blood pressure

**Table 4** summarises changes in blood pressure. SBP showed a significant main effect of time [F(1, 54) = 8.279, p = 0.006, partial η^2^ = 0.133] and a significant group × time interaction [F(2, 54) = 3.391, p = 0.041, partial η^2^ = 0.112]. *Post hoc* analysis indicated that SBP decreased from baseline in both the morning (p = 0.022) and evening (p = 0.003) exercise groups. For DBP, a significant main effect of time was observed [F(1, 54) = 7.435, p = 0.009, partial η^2^ = 0.121], whereas the group × time interaction was not significant (p = 0.089). MAP also showed a significant time effect [F(1, 54) = 8.909, p = 0.004, partial η^2^ = 0.142] and a significant group × time interaction [F(2, 54) = 3.262, p = 0.046, partial η^2^ = 0.108]. *Post hoc* analysis showed decreases from baseline in both the morning (p = 0.012) and evening (p = 0.004) exercise groups, with lower post-intervention MAP in the evening than in the control group (p = 0.012). Exploratory analyses limited to the two exercise groups revealed no significant group × time interactions for SBP, DBP or MAP (all p > 0.05).

**Table 4:** Blood pressure outcomes at baseline and post-intervention.

Outcome		Time		Group		Time		Interaction (time×group)	
	Group	Pre	Post	p	Partial η^2^	p	Partial η^2^	p	Partial η^2^
SBP (mmHg)	CON	130.61 ± 20.81	132.00 ± 21.16						
	ME	134.70 ± 14.31	127.50 ± 17.34*	0.491	0.026	0.006	0.133	0.041	0.112
	EE	130.47 ± 17.92	120.68 ± 16.11*						
DBP (mmHg)	CON	79.22 ± 11.14	79.89 ± 10.80						
	ME	78.70 ± 9.26	73.10 ± 9.44	0.110	0.078	0.009	0.121	0.089	0.086
	EE	77.21 ± 7.17	71.16 ± 7.25						
MAP (mmHg)	CON	96.35 ± 13.60	97.26 ± 13.60						
	ME	97.37 ± 8.96	91.23 ± 10.83*	0.237	0.052	0.004	0.142	0.046	0.108
	EE	94.96 ± 9.71	87.67 ± 9.11*#						

Data are presented as mean ± SD. Results are based on two-way repeated-measures ANOVAs comparing groups (ME vs EE vs CON) in both phases (pre vs. post), followed by LSD *post-hoc* tests. #means a significant difference compared with the control group at post-intervention; *indicates a significant within-group difference before and after the intervention (p < 0.05).

SBP, Systolic Blood Pressure; DBP, Diastolic Blood Pressure; MAP, Mean Arterial Pressure.

## 4 Discussion

The primary findings of this study were that both morning and evening exercise improved FMD and reduced SBP, MAP, and baPWV in older adults with T2DM, while plasma NO levels increased significantly from baseline only in the evening exercise group. Compared with the control group, the evening exercise group had higher post-intervention FMD and plasma NO levels and lower MAP. Exploratory analyses comparing the two exercise groups showed a greater baPWV reduction and a tendency towards a greater NO increase with evening exercise. These findings suggest that evening exercise may confer additional vascular benefits in this population, particularly in reducing arterial stiffness.

Our findings are consistent with previous studies showing that exercise can improve endothelial function in individuals with T2DM (10, 34). FMD is a recognised independent predictor of future cardiovascular events, with evidence suggesting that a 1% increase in FMD is associated with an approximately 13% reduction in the risk of cardiovascular events (35). In our study, FMD increased by an average of 1.45% among participants in the two exercise groups, indicating potentially clinically relevant improvements in endothelial function.

Evidence from long-term interventions comparing morning and evening exercise remains limited. Shen et al. (24) reported that, following a 12-week aerobic training intervention in sedentary adult men, arterial stiffness improved in both the morning and evening exercise groups, and several carotid haemodynamic measures also improved in the evening group. Unlike this aerobic-only intervention, our study adopted a combination of aerobic and resistance exercise and examined vascular adaptations at multiple levels, including endothelial function, arterial stiffness, and circulating NO metabolites. Although FMD increased numerically more in the evening group (1.67%) than in the morning group (1.25%), the exploratory analysis identified no significant group × time interaction.

Plasma NO levels increased significantly only following evening exercise, whereas no significant change was observed in the morning group. An exploratory analysis suggested a tendency towards a greater increase with evening exercise (group × time interaction, p = 0.051). Plasma NO responses showed considerable inter-individual variability, which may partly reflect heterogeneity in vascular status within this population. Plasma NO and FMD provide complementary biochemical and functional perspectives on vascular adaptation. The NO findings raise the possibility that exercise timing may influence circulating NO responses to training.

Diurnal variation in autonomic and vascular regulation provides a physiological context for these potential effects of exercise timing. Jones et al. (36) observed that acute morning exercise was associated with a higher brachial artery shear rate and blood flow velocity without a compensatory increase in arterial diameter and was accompanied by elevated MAP, suggesting enhanced sympathetic vasoconstrictor activity in the morning that may elevate cardiovascular risk. This aligns with human circadian patterns, in which sympathetic activity peaks early in the morning, contributing to increased vascular tone and blood pressure (37). Therefore, the vascular benefits of morning exercise may be attenuated by a circadian-driven increase in sympathetic vasoconstrictor activity, which may impair vasodilatory responses. In contrast, evening exercise occurs during periods of lower sympathetic tone, potentially facilitating greater vasodilation and vascular adaptations.

Circadian patterns in vasoactive factors may also influence responses to exercise performed at different times of day. NO levels tend to be lower in the early morning and higher in the evening (38), while FMD is similarly attenuated in the morning and increased later in the day (39). These patterns may create a more favourable vascular environment for postexercise vasodilation. However, most studies examining time-of-day effects on endothelial function were based on acute exercise interventions, with inconsistent findings. Jones et al. (22) reported no significant change in FMD following morning exercise but a reduction following afternoon exercise, whereas Ballard et al. (23) observed no significant postexercise change in FMD at either time point. However, acute exercise interventions have some limitations. First, the effects of acute exercise on FMD remain controversial, with some studies showing improved FMD postexercise (40–45) and others reporting significant reductions (45–47). Moreover, conducting acute interventions at different times of the day inevitably places FMD assessments in varying circadian phases, which may confound the interpretation of exercise-induced changes due to underlying diurnal fluctuations in endothelial function. Notably, our study controlled for this confounding factor by ensuring consistent timing of pre- and post-intervention endothelial assessments, allowing for a more accurate evaluation of chronic vascular adaptations induced by time-of-day-specific exercise.

Arterial stiffness refers to the reduced capacity of the arteries to expand and contract with pressure changes (48). PWV, the most commonly used clinical marker of arterial stiffness (49), increases with vascular rigidity (50). Ageing and T2DM accelerate arterial wall remodelling and elastic fibre degradation, leading to an elevated PWV (51). In our study, baPWV declined in both exercise groups, with mean reductions of 89.2 cm/s in the morning group and 221.1 cm/s in the evening group after the 12-week intervention. The exploratory analysis showed a significant group × time interaction (p = 0.034), indicating a greater reduction with evening exercise. Exercise promotes arterial compliance by enhancing blood flow and increasing shear stress on endothelial cells (11), thereby stimulating endothelial nitric oxide synthase (eNOS) phosphorylation and NO production (52). Endothelium-derived NO plays a key role in modulating vascular tone and arterial elasticity (53, 54) and may therefore contribute to exercise-induced improvements in arterial compliance. Together with the tendency towards a greater increase in plasma NO, the larger baPWV reduction following evening exercise suggests a possible contribution of NO-related vascular pathways to these arterial adaptations.

Both exercise groups also showed significant reductions in SBP and MAP. Post-intervention MAP was lower in the evening exercise group than in the control group. Previous studies by Brito et al. (55, 56) reported greater blood pressure reductions with evening aerobic exercise in patients with hypertension. However, no significant differences in blood pressure changes between morning and evening exercise were detected in the present study. This variation may partly reflect differences in clinical characteristics and exercise modality.

To the best of our knowledge, this is the first randomised controlled trial to compare the long-term effects of morning and evening combined aerobic and resistance exercise on endothelial function, arterial stiffness, and blood pressure in older adults with T2DM. A key strength of this protocol is the consistent timing of the vascular function assessments before and after the intervention, which minimises the potential confounding influence of circadian variations on outcome measures.

This study has some limitations. First, owing to practical constraints, we did not include an afternoon exercise group. Whether afternoon exercise confers different vascular benefits compared with morning or evening sessions remains to be determined. Second, baseline lipid-lowering medication use was imbalanced across groups. Although this imbalance likely arose by chance following randomisation, it may have influenced the observed between-group vascular responses. Third, although the participants were asked to maintain their usual lifestyle and medication regimens, dietary intake was not strictly controlled, and sleep, physical activity outside the prescribed exercise sessions, and medication timing were not monitored during the intervention. Additionally, shear rate was not assessed during FMD measurement because our ultrasound system did not permit simultaneous recording of brachial artery diameter and blood velocity after cuff deflation. Future studies should monitor dietary intake, sleep, physical activity and medication timing throughout the intervention and include longer follow-up to determine whether the observed vascular adaptations and potential differences related to exercise timing are sustained.

## 5 Conclusions

This study compared the effects of morning versus evening exercise on vascular function in older patients with T2DM following a 12-week combined aerobic and resistance training intervention. Both the morning and evening exercise groups showed improved vascular function; however, the evening exercise group showed a greater reduction in baPWV and a numerically greater increase in plasma NO. These findings suggest that exercise timing may influence vascular adaptations in older patients with T2DM, with evening exercise potentially providing additional benefits, particularly for arterial stiffness.

## Data Availability

The raw data supporting the conclusions of this article will be made available by the authors, without undue reservation.
